# Epidemiological Features of Highly Pathogenic Avian Influenza in Cameroon

**DOI:** 10.1155/2019/3796369

**Published:** 2019-01-27

**Authors:** Marc K. Kouam, Honorine N. Tchouankui, Arouna Njayou Ngapagna

**Affiliations:** ^1^Department of Animal Production, Faculty of Agronomy and Agricultural Sciences, P.O. Box 188, Dschang, Cameroon; ^2^Center for Research on Filariasis and Other Tropical Diseases (CRFilMT), P.O. Box 5797, Yaoundé, Cameroon; ^3^Unit of Veterinary Public Health and Clinical Sciences, Faculty of Veterinary Medicine, Universite des Montagnes, Cameroon

## Abstract

The epidemiology of avian influenza is unknown in Cameroon despite the two outbreaks that occurred in 2006 and 2016-2017, respectively. In order to fill the gap, an attempt was made to provide some basic information on the epidemiology of highly pathogenic avian influenza in Cameroon. Thus, data were collected from follow-up reports of the second HPAI outbreaks prepared by the veterinary health officials of Cameroon and sent to the World Organisation for Animal Health (OIE). Two HPAI virus strains (H5N1 and H5N8) turned out to occur, with H5N1 virus involved in the Center, South, West, and Adamawa regions outbreaks and H5N8 involved in the Far North outbreak only. The affected hosts were the laying hens, backyard chickens, turkeys, guinea fowls, ducks, broiler and layer breeders, and geese for the H5N1 virus and the Indian peafowl (*Pavo cristatus*), pigeon, ducks, backyard chickens, and guinea fowls for the H5N8 virus. The first outbreak took place in Mvog-Betsi poultry complex in the Center region on the 20^th^ May 2016 and spread to other regions. The mortality rate varied from 8% to 72% for H5N1 virus and was 96.26% for the H5N8 strain in Indian peafowl. No human case was recorded. The potential supporting factors for disease dissemination identified on the field were the following: poultry and eggs dealers moving from one farm, market, or town to another without any preventive care; poor biosecurity measures on farms and live poultry markets. After the first HPAI H5N1 virus outbreak in 2006, the second HPAI outbreak ten years later (2016-2017) involving two virus strains is a cause of concern for the poultry industry. The Cameroon Epidemio-Surveillance Network needs to be more watchful.

## 1. Introduction

Highly pathogenic avian influenza (HPAI) is a zoonotic viral disease of birds, swine, and man occurring worldwide [1, 2]. HPAI is caused by influenza type A viruses, especially subtypes H5 and H7 [3]. Influenza viruses of these subtypes are the most important causes of HPAI outbreaks in Europe, Asia, Africa, and the Pacific where they cause high mortalities and poultry destruction on poultry farms and wildlife [4, 5]. Among the viruses, the H5N1 strain has been reported to be the most circulating one in Africa [2] and the most important threat to public health [2, 4]. In infected poultry, the symptoms of H5N1 infections are of wide range. These include [4] sudden death, high mortality, weakness, and recumbency; others ranged from nasal discharges, dyspnea, coughing, sneezing, diarrhea, shank hyperemia and hemorrhage, inability to stand, ataxia, and torticollis; in layers, egg structural abnormalities such as shell-less egg, white-colored eggs, and soft eggs occurred; lesions observed in the circulatory system included congestion, cyanosis of comb and wattle comb and wattle edema, and facial and subcutaneous edema. At necropsy, airsacculitis and pneumonia within the respiratory system or petechiation to ecchymoses of the proventricular and intestinal mucosa with resultant enteritis in the gastrointestinal system may be noticed [4]. Integumentary system lesions (mainly cyanosis, edema, and ecchymotic hemorrhages) as well as inflammatory, degenerative, and necrotic lesions in the musculoskeletal system may also be seen at necropsy [4].

For human infections with avian influenza H5N1 virus, the disease shows a range of clinical manifestations from fever and cough to severe pneumonia, distressed breathing, shock, and death [6, 7]. Gastrointestinal clinical signs such as nausea, vomiting, and diarrhea have also been reported [8].

So far, two strains have been reported in Cameroon: the first (H5N1) in both the 2006 and 2016-2017 HPAI outbreaks, and the second (H5N8, clade 2.4.4.4 ) in the 2016-2017 epidemic [2, 9]. However, basic epidemiological data on HPAI in Cameroon are unavailable or scattered. Thus, the main objective of this study was to present some epidemiological features of HPAI in Cameroon. More specifically, the study is aimed at describing the mortality rate, the main hosts, and the distribution of the disease during the most recent epidemics.

## 2. Materials and Methods

### 2.1. Study Area

This descriptive cross-sectional study was conducted in the regions of the country where the outbreaks occurred. These include the Central, Southern, Western, Adamawa, and Far North regions of Cameroon.

### 2.2. Data Collection

The data were excerpted from follow-up reports of the 2016-2017 HPAI outbreaks sent by the veterinary health officials of Cameroon to the World Organisation for Animal Health (OIE). These reports were prepared starting from the first up to the last outbreak, by the officials from different governmental bodies and institutions including the Cameroon Epidemio-Surveillance Network (Reseau d'Epidemio-Surveillance au Cameroun [RESCAM]) of the Ministry of Livestock, Fisheries and Animal Industries (MINEPIA), and the Direction of veterinary services (Direction des Services Vétérinaires [DSV]). For the detection of disease in animals, cloacal and tracheal swaps of animals in different disease sources were collected and examined by the National Veterinary Laboratory (LANAVET), the Centre Pasteur du Cameroun (CPC), and the military laboratory (Centre de Recherche pour la Santé des Armées [ CRESAR]). Human individuals exposed to avian influenza were followed-up; thus, blood samples were also collected and analyzed by the CPC from humans that had been in contact with diseased birds or bird corpses suspected to have died from HPAI. The diagnostic test performed in all these analyses was the real time PCR (RT-PCR) as described by Hoffman et al. [10].

## 3. Results

### 3.1. Virus Strains, Animal Host, and Disease Distribution

Two strains (H5N1 and H5N8) were detected. H5N1 virus was detected in broilers, laying hens, backyard chickens, turkeys, guinea fowls, ducks, broiler and layer breeders, and geese. H5N8 virus occurred in the Indian peafowl (*Pavo cristatus*), pigeon, ducks, backyard chickens, and guinea fowls.

**Figure 1:**
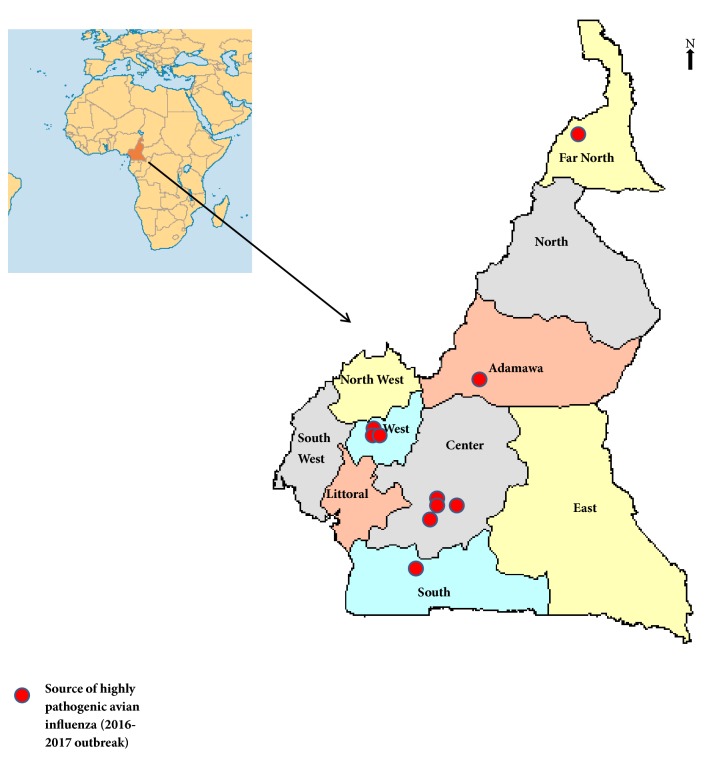
Map of Cameroon showing the sources of highly pathogenic avian influenza virus outbreak in Cameroon. (The H5N1 outbreak occurred in 2016 in the Center, South, West, and Adamawa regions while the H5N8 outbreak took place in the Far North Region in 2017.)

H5N1 virus was found to be the aetiologic agent of HPAI in all the foci of Adamawa, South, Center, and West region of Cameroon (Figure 1). H5N8 was found only in the Far North (Figure 1).

Blood samples from 481 humans exposed to the disease from four regions were tested. None of the tested samples was HPAI-virus positive. Out of this total number, only 136 (28%) were tested for a second round, 7 days after the first round but still none of the samples was positive. No case of human contamination has been reported so far.

### 3.2. Mortality Rate

The mortality rate due to strain H5N1 varied from 8 to 72% in the West and Adamawa regions, respectively (Table 1). For H5N8 virus, the mortality rate in Indian peafowls was 96.26% (103 out of 107 birds).

### 3.3. Mechanism of Distribution and Risk Factor of the HPAI Virus

**Figure 2:**
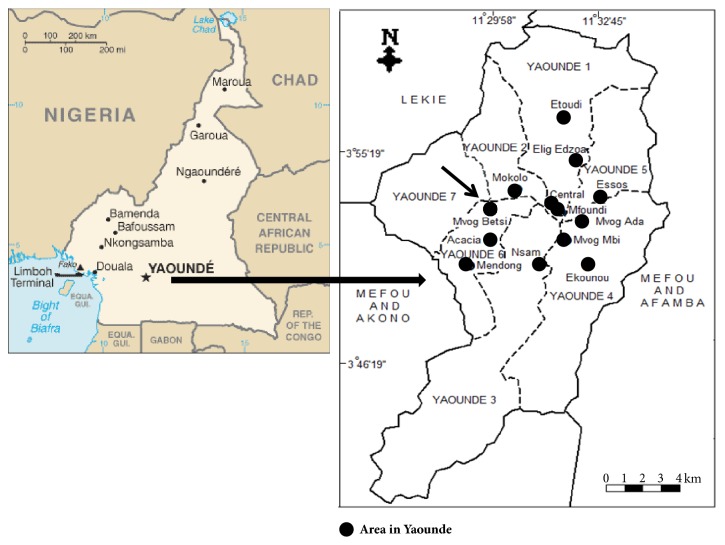
Map of Yaounde showing Mvog-Betsi area (arrow) where the first highly pathogenic avian influenza outbreak took place in May 2016.

The first outbreak of HPAI was reported in Mvog-Betsi poultry complex in Yaounde (Center region) (Figure 2) on 20^th^ May 2016 followed by successive other outbreaks in markets and farms still in the Center region, and in the South, West, Adamawa, and Far North regions as well. The potential supporting factors for disease dissemination identified on the field were the following: poultry and eggs dealers moving from one farm to another, from one town to another, or from markets to farms without any hygienic protection; poultry collectors acting the same way as the dealers; a very poor biosecurity level on farms and at market places.

## 4. Discussion

The first official report of the most recent epidemic of HPAI was issued on 27^th^ May 2016. However, the outbreak was suspected to have occurred much earlier than officially stated for some reasons which include the important traffic for trading purposes between Cameroon and Nigeria, the porous borders of the country, and the contact between wild birds and backyard chickens. All these conditions would have eased the introduction of the avian influenza virus in the country from areas of the world where the epidemic was ongoing, such as West Africa [2, 11]. This is confirmed by the fact that the strains occurring in Cameroon outbreaks were the same strains reported in these areas [2, 12]. This is also supported by the detection of the H5N8 virus in a wild bird, the Indian peafowl.

The number of sources varied per region, and each included the poultry markets and farms. The spread of the disease can be explained by many factors at farm, market, and transportation level. At farm level, these factors include the poor biosecurity practices inside and around the farm [13], the low level of technicality in management practices, the low education level of the staff and employees, and the permanent rotation of the personnel observed [14, 15]. At market level, the origin of fowls is unknown or untraceable, making any attempt to trace back the origin of disease quite impossible. For this reasons, some measures such as disinfection and closure of the market places, and ban of poultry and poultry by-products trading in affected markets helped to stamp out the disease and to stop further spread to other sites. Regarding transportation, lack of disinfection of the transportation means of live birds and eggs (trucks, lorries, vans) from one farm to another, or from one market to another, might explain why the disease easily spread in the country.

The higher number of farms in the South region was probably due to its proximity with the Center region where the first outbreak occurred. Indeed supply of chicks, eggs, feedstuff, and any farming tool to the South region is exclusively carried out from the Center region.

The mortality rates observed in birds were similar to those documented in Nigeria which were reported to vary between 11.11% and 73.92% [4]. This might be explained by the common strains occurring in both countries, as well as the low biosecurity practices reported in the two countries [13, 16].

Various groups of poultry were affected in this recent avian influenza epidemic comprising poultry from commercial farms, backyard farming systems, and exotic farms, indicating important economic losses in poultry industry in the country. The losses were induced by the high poultry mortalities on farm due to HPAI, but also from the high number of live poultry that were destroyed as a preventive measure in affected farms without any compensation to farmers. Though depopulation is known as an efficient mean to contain the spread of HPAI [17], it is advisable, in view of the tough economic conditions of the country, to prevent the disease by using vaccines as is done elsewhere [2].

Human infections with avian influenza are mostly known to be due to H5N1 virus [7] which has been reported in the 2006 and 2016-2017 outbreaks in Cameroon. However, no human case has been detected in Cameroon, which might be explained by the fact that the strains circulating in the country are not adapted to humans, due to the high genetic diversity in influenza A viruses [18, 19].

In conclusion, the study showed that the most recent epidemic of HPAI in Cameroon that occurred from 2016 to 2017 was caused by two virus strains that led to high mortalities of poultry in commercial, backyard, and exotic farms type. Though the disease was geographically distributed in five over ten regions of the country, how the disease was introduced in the country is unknown. More genetic and epidemiological data may help clarify by which means, when, and from where the viruses were introduced in Cameroon. The occurrence of a second strain (H5N8) in addition to the previous one (H5N1) that first occurred in 2006, is an indication that the poultry sector needs to be cautious and on permanent watch to avoid a third outbreak.

## Figures and Tables

**Table 1:** Number of sources and mortality rate of the 2016 highly pathogenic avian influenza H5N1 outbreak in Cameroon.

**Region**	**Total number of sources**	**N**	**Total number of deaths**	**Mortality rate (**%**)**

**Center**	06	41 844	16 345	39,06
**South**	08	12 819	4 086	31,87
**West**	03	46 828	4 189	8,94
**Adamawa**	01	66	48	72,72
**Total**	18	101 557	24 668	24,28

N: total number of poultry in affected sites (live-poultry markets, farms, backyards); the fowls that did not die were stamped out for control purposes; the affected sites were the live-poultry markets and farms for the Center, South, and West sources and the backyards for the Adamawa source.

## Data Availability

The data used to support this work are included in the manuscript.
